# The relationship between psychology practice and complementary medicine in Australia: Psychologists’ demographics and practice characteristics regarding type of engagement across a range of complementary medicine modalities

**DOI:** 10.1371/journal.pone.0285050

**Published:** 2023-05-04

**Authors:** Carrie Thomson-Casey, Erica McIntyre, Kris Rogers, Jon Adams

**Affiliations:** 1 School of Public Health, Faculty of Health, University of Technology Sydney, Sydney, Australia; 2 Faculty of Health, Southern Cross University, Gold Coast, Australia; 3 Institute for Sustainable Futures, University of Technology Sydney, Sydney, Australia; Singapore General Hospital, SINGAPORE

## Abstract

**Introduction:**

Many people with mental health problems utilise a range of complementary medicine (CM) practitioners, products, and practices. Psychologists are likely to consult with clients who are seeking and using CM, in some form, as part of their wider mental health treatment. The aim of this research is to determine how much, and in what ways, Australian psychologists recommend CM products and/or practices, and/or initiate referrals to CM practitioners as part of their clinical practice and to explore if these behaviours have any association with the characteristics of the psychologist or their wider practice.

**Methods:**

Survey data was collected from psychologists in clinical practice who self-selected to participate between February and April 2021. Participation in the study was via an online 79-item questionnaire exploring core aspects of CM engagement in psychology clinical practice.

**Results:**

Amongst the 202 psychologists who completed the survey, mind/body approaches (90.5%) were the most recommended CM and cultural/spiritual approaches the least recommended CM (7.5%). Participants also reported referring to CM practitioners with naturopaths the most common focus of their referrals (57.9%) and cultural and spiritual practitioners the least common focus of their referrals (6.69%). Our analysis shows the demographic and practice characteristics of a psychologist are generally not predictors of a psychologist’s engagement with CM in their clinical practice.

**Conclusions:**

Substantial numbers of psychologists recommend CM products and practices and/or refer clients to CM practitioners. Alongside subjecting CM interventions for mental health to an evidence-base assessment, the broader discipline of psychology needs to also consider psychologist engagement with CM in clinical practice in order to help ensure cultural-sensitivity, client safety and client choice.

## Introduction

Psychologists are likely to have clients who choose complementary medicine (CM), in some form, as part of their wider mental health treatment [1, 2]. CM refers to health care products, services and practices, that are “not part of a country’s own traditions or conventional medicine and are not fully integrated into the dominant health care system” [3] and often includes a wide range of products, services and practitioner types that can vary with cultural and political context [4–7].

CM use is high among the general population. For example, an Australian study of the general population found 63.1% of participants used some form of CM and 52.8% had consulted a CM practitioner (e.g., massage therapist, naturopath, osteopath) in the last 12 months [8]. Consumers choose CM for a range of reasons including: alignment with cultural and personal beliefs; an expectation of benefit; dissatisfaction with conventional approaches; to help address side effects caused by conventional medication use; to address symptoms related to severe or complex illness; and/or as a preference for a holistic approach to their care [9–11].

CM use is also high among people with mental health problems who have been found to utilise different types of CM practitioners, including naturopaths and massage therapists, as well as a range of CM products and practices [7, 8, 12, 13]. An Australian study of adults diagnosed with a mental health disorder reported 42.4% consulted a CM practitioner, 56.9 % used a complementary medicine product and 23 % used a complementary medicine practice in the previous three years [14]. Similarly, another Australian study found 21.3% of participants, who were middle aged women with a diagnosis of depression, had consulted at least one CM practitioner in the last 12 months [13].

There is emerging evidence for some CM approaches in addressing mental health symptoms, such as nutraceuticals (e.g., omega 3 fatty acids, probiotics, and zinc) [15], nutrition (e.g., the Mediterranean diet) [16], herbs (e.g., St John’s wort, saffron) [15, 17–19] and probiotics [20] for depression. Manual therapies and movement approaches also demonstrate benefit for people experiencing stress, anxiety and depression [21–24]. Although these CM approaches show promise as part of preventative and adjunct treatments for mental health, it is acknowledged there is much more research needed to further critically appraise the efficacy of specific CM [25–27].

There has been commentary, beyond and within psychology, that criticises the field for what has been seen as a monocultural Westernised approach to mental health care that diminishes the relevance of CM and traditional healing approaches associated with specific cultural and ethnic groups [28, 29]. Meanwhile, others have highlighted the importance of psychologists embracing culturally sensitive practice, which may include acknowledgement of a clients’ preference for CM use [30–32].

Consistent with the latest World Health Organisation Traditional Medicine Strategy [33], CM has been integrated into a number of health care settings [34, 35] including those providing mental health services [36–40]. CM integration into health care settings may take a variety of forms facilitated through a range of practice circumstances and practitioner networks [41] and CM integration in the context of psychology practice, and for purposes of this study, refers to a psychologist engaging with CM in some form in their clinical practice as part of a client’s mental health treatment planning. Such integration can be via: *discussing* CM with a client in their care (e.g., asking questions about a client’s CM use), *recommending* CM to a client in their care (e.g., suggesting a client attend a yoga class for relaxation), *referring* a client in their care to a CM practitioner (e.g., verbal or written referral to Western herbal medicine practitioner), the psychologist directly practising and *applying* CM to a client in their care (e.g., explicit dietary instructions to improve nutrition in the context of evidence-based nutritional psychiatry), and/or a combination of these options above.

Some CM approaches have been integrated into psychology practice [42–44]. For example, mindfulness, emotional freedom technique (EFT), and eye movement desensitisation and reprocessing (EMDR), all previously considered fringe or beyond the field of psychology, are now considered evidence-based psychology interventions [45, 46]. Similarly, nutritional psychiatry has emerged as a significant paradigm in mental health care [27, 47, 48] and although not traditionally part of psychologists’ tertiary education [49], psychologists are increasingly engaging with this approach [50]. There is limited research describing the referral practices of psychologists to CM practitioners, which may be a reflection of limited guidelines for psychologists on how they might engage with CM in their practice [51].

As novel approaches to mental health care emerge psychologists have sought additional training in CM, are subsequently more inclined to engage with CM, and are discerning about recommending and referring to CM practitioners [30, 52–54]. An Indonesian study of clinical psychologists reported approximately 73% were recommending CM to their clients and 39% were referring to a CM practitioner [55]. Reasons psychologists choose to engage with CM in their clinical practice include positive experiences with personal use of CM, receiving education in CM, and wanting to offer a holistic service to clients that includes CM [49, 54, 56]. However, some psychologists express concern about a broader lack of education and guidelines for the integration of CM within psychology [27, 52, 54, 57, 58]. Overall, the acceptance of CM within the discipline of psychology remains contested [58–61]—a situation not dissimilar to that for medicine and other health disciplines [62, 63].

Previous work suggesting psychologists in Australia are favourable toward CM [64–66] has invariably included focus upon psychology students and interns, academic psychologists, psychologists from other countries, and other mental health professionals. In contrast, the research reported here focusses exclusively on psychologists in clinical practice to examine how often and in what ways these grass-roots practitioners recommend a range of CM products and/or practices to their clients, and/or refer their clients to a range of CM practitioners.

## Methods and materials

### Aim

The aim of this research was to determine how much, and in what ways, Australian psychologists recommend CM products and/or practices, and/or initiate referrals to CM practitioners as part of their clinical practice and to explore if these behaviours have any association with the characteristics of the psychologist or their wider practice.

### Study design

A survey was distributed online to Australian psychologists who were fully registered and working in a clinical practice setting at time of recruitment (between February and April of 2021). Email invitations to participate in the study were sent to psychologists whose contact details were collected from their publicly available websites. Recruitment emails contained information about the study, consent forms, and a link to complete the survey online. A reminder email was sent to psychologists four weeks after the initial invitation email. An advertisement inviting psychologists to participate in the research was also placed on two psychology professional association websites (Australian Association of Psychologists Incorporated and the Australian Psychological Society) and on relevant social media sites including Twitter, LinkedIn, and Facebook. Participants accessed the survey via an anonymous link embedded in the website advert, or social media post, and were directed to the participant information and consent form via the link. The open survey was completed online via Qualtrics software, Version 2021 [67]. The information page at the beginning of the survey included project details such as ethical approval, data protection, and voluntary participation. The information page also served as the participant consent form. Participants indicated their written consent after reading the information and consent page and clicking on the button confirming their agreement to proceed with the survey. Upon completion of the survey participants were invited to supply their email address to enter a prize draw to win a $250 gift voucher. Ethical approval was attained from UTS Human Research Ethics Committee [ETH20-5138].

#### Sample

The survey was distributed to 1479 Australian psychologists working in clinical practice at the time of research. All psychologists in Australia (n = 34,872) are considered to hold general registration, which enables them to use the title of psychologist [68]. Some psychologists with additional tertiary training in psychology may also hold an area of practice endorsement (AoPE) enabling them to use a restricted title (e.g., clinical psychologist). These AoPE titles are clinical neuropsychologist, clinical psychologist, community psychologist, counselling psychologist, educational and developmental psychologist, forensic psychologist, health psychologist, organisational psychologist, and sport and exercise psychologist. A psychologist with an AoPE title has general registration plus an AoPE. To clarify, a psychologist with general registration, without an AoPE (i.e., psychologist), can work in clinical practice settings; however, working in clinical practice does not mean a psychologist is necessarily a clinical psychologist. All psychologists (psychologists with general registration and those psychologists with general registration plus an AoPE) were eligible to participate in the study; however, only responses from those psychologists who work in a clinical practice setting (e.g., inpatient hospital, private practice) directly with clients were included in the data analysis for this study.

The initial screening question asked participants if they were a psychologist undertaking work as a psychologist. Participants who selected “No” were redirected out of the survey. Prior to conducting the analyses, raw survey data were screened for any missing or incomplete responses and duplicate IP addresses. While there were no duplicate IP addresses, during this process, nine cases were removed as the data (responses) were incomplete. After removal of the nine cases, 222 cases were included in the initial analyses which identified significant outliers. On review the outlier responses were mostly from cases who did not work in clinical practice settings. These cases were removed resulting in 201 participants in the final data set. The original sample size was planned to be 400, based on achieving a 0.10 confidence interval width on estimates of prevalence of binary questionnaire items. As noted above, we were able to recruit 231 participants, of which 201 passed the inclusion criteria and were used in this study. With this sample size we are able to estimate a confidence interval for the prevalence of a single binary item with a CI width of 0.14; or compare a continuous or binary variable between two equally sized groups with 0.8 power and an effect size of 0.39 (Cohen’s D).

#### Instrument

The construction of survey items was informed by previous literature on psychologist engagement with CM to produce survey items that best captured the ways psychologists might be engaging with CM, including the types of CM products and/or practices they had ever recommended and the types of CM practitioners they had ever referred to. The 79-item questionnaire aimed to examine the extent and ways in which psychologists consider CM relevant and/or appropriate to (their) psychology practice, their clients, and the treatment of mental health problems. Participants were also provided with a definition of CM similar to the one provided above and in line with the World Health Organisation Traditional Medicine Strategy [33]. The survey collected participants’ demographics and practice-related information, any relevant qualifications attained or professional memberships outside of psychology, participants’ perspectives on their scope of practice in Australia in relation to any kind of adjunctive additional health qualifications, and finally participants’ perspectives and behaviours regarding engagement with CM in clinical practice in Australia. Prior to recruitment the survey was tested for face validity and functionality by three PhD students from psychology adjacent fields. Changes were made to provide clearer definitions and reduce repetitive questions. Based on feedback from the PhD students who tested the survey, the time required to complete the survey was approximately 15 minutes. Where relevant this paper adhered to the CHERRIES checklist for reporting results of internet e-surveys [69, 70].

#### Demographics

The survey collected data regarding each participant’s year of birth, gender identity, and the predominant state in which they practise. Participants were also asked to provide practice characteristics, such as AoPE, their work setting (solo or group setting), and years in practice as a psychologist.

#### Additional qualifications

The survey collected data on participants’ tertiary qualifications in addition to their psychology qualification (i.e., business, criminal justice/criminology, dietetics, education, exercise physiology, law, medical, nursing/midwifery, physiotherapy) and CM related professional qualifications (naturopathy, nutrition, traditional Chinese medicine, Western herbal medicine, yoga instructor) as well as the options of “No” or “Other”. If a psychologist selected “Other” they could then add text to describe their additional qualification. If the psychologist indicated they had an additional qualification they were asked further questions in relation to that additional qualification including: highest level of education in that qualification, if they have separate insurance and/or professional membership for that qualification, and whether they integrate that additional qualification into their psychology practice and treatment planning with clients.

#### Psychologists’ scope of practice

Psychologists were asked to indicate their level of personal agreement/disagreement with regards to statements describing psychologists using practices/treatments from an additional health-related qualification with their psychology clients (e.g., a psychologist treating a client from two separate qualifications, such as a psychologist and a dietitian). Questions on this topic explored attitudes toward psychologists who utilised an additional health-related qualification in the context of: treatment planning; communication with clients, client outcomes, and impacts on the scope of psychology practice and psychology as a discipline if a psychologist incorporated a second health related discipline into their practice.

#### Attitudes towards CM in the context of psychology practice

The last section of the survey invited psychologists to rate their personal agreement/disagreement with statements relating to psychologist engagement with CM products, practices and practitioners. Likert scales were used to record participant attitudes toward CM (six response choices ranging from strongly agree to strongly disagree) and are reported as measured. Participants also reported the types of CM products and/or practices they recommend to clients, and the types of CM practitioners they have referred to at any time as part of their psychology practice.

#### Data analysis

IBM SPSS Statistics Premium Edition Version 27 (Armonk, New York, IBM Corp) was used to analyse the data. Descriptive statistics were used to determine the percentages and frequencies. Chi-square analysis was used to assess associations between categorical demographic and practice characteristics and CM engagement related variables (*recommending* CM products and/or practices, and/or *referring* to CM practitioner types). A Firth’s correction for logistic regression was employed due to small cell sizes and to address the possibility of separation of data [71]. Descriptive tables reporting the percentage of psychologists who recommend CM products and practice, as well as those who refer to CM practitioners is provided in the appendix. A complete-case analysis was used to deal with item non-response.

Psychologists have general registration; however, some also hold an AoPE. There were an adequate number of participants to create groups for general psychologists and AoPE clinical psychologists, but the remaining individual AoPE categories (community psychology, counselling psychology, educational and developmental psychology, forensic psychology, health psychology) were small (cell size < 5). An additional category was created for these psychologists called *other AoPE*.

For analysis purposes, the types of CM products and/or practices were categorised into six groups as informed by previous literature [5, 72, 73] and consistent with the definition of CM used in this study: mind/body approaches (hypnotherapy, meditation, yoga), movement approaches (exercise and movement-based activities, such as walking), ingestive therapies (herbal medicine, probiotics, vitamin and nutrition supplements), dietary changes, and manual approaches (acupuncture and massage). The sixth category, cultural and spiritual approaches, included participant’s free text responses indicating recommending or referring to music, creative arts, prayer and spirituality, and Aboriginal and Torres Strait Islander traditional healing.

The types of CM practitioners were categorised into six groups that were informed by previous literature [72, 73] and consistent with the definition of CM used in this study. For the purpose of this this study the six practitioner categories included: mind/body practitioners (i.e., hypnotherapists and yoga teachers), movement practitioners (i.e., exercise and movement trainers and/or coaches), practitioners who predominantly prescribe ingestibles (i.e., naturopaths, herbalists, and traditional Chinese practitioners), prescribes nutrition (i.e., nutritionists), and manual (i.e., acupuncturists, chiropractic, massage therapists). As the number of participants referring to Aboriginal and Torres Strait Islander traditional healers was small, a new category of cultural and spiritual practitioners was created that incorporated participant free text responses which indicated they refer to music, creative arts, kinesiology and energy practitioners.

## Results

Of the completed surveys, 65% (n = 134) were completed via email invitation link and the remaining 35% (n = 68) accessed the survey via website or social media link as described above. The completion rate for opened surveys was 77%.

### Participant characteristics

The study sample (n = 202) comprised 165 women (81.6%, Table 1), 36 men (17.8%) and one person who identified as other (0.5%). The mean age of participants was 48 years (*M* = 48, *SD* = 26). All Australian states and territories were represented within the sample, with highest representation from New South Wales (n = 65) and Queensland (n = 64) and the lowest from Northern Territory (n = 1). Most psychologists identified as holding the AoPE of clinical psychologist (n = 79) or a psychologist with general registration (n = 76). Participants also came from other AoPE including counselling psychologist (n = 25), forensic psychologist (n = 8), health psychologist (n = 7), educational and developmental psychologist (n = 6), and one community psychologist. Solo practice was the most common work setting reported among participants (n = 137). The highest proportion of participants reported having 11 to 20 years of clinical experience (n = 72) and the lowest proportion (n = 31) reported having 31 plus years of clinical experience.

**Table 1:** Psychologist characteristics. Psychologist demographic and practice characteristics, number (n) and percent of Area of Practice Endorsement (%).

	All (n = 202)		General psychologist (n = 76)		Clinical psychologist (n = 79)		Other AoPE psychologist (n = 47)	
	n	%	n	%	n	%	n	%
**Gender**								
*Female*	165	81.7	62	81.6	68	86.1	35	74.5
*Male*	36	17.8	14	18.4	10	12.7	12	25.5
*Other*	1	0.5	0	0.0	1.0	1.3	0	0.0
**Age (years)**								
*18 to 35*	20	9.9	7	9.2	13	16.5	0	0.0
*36 to 50*	66	32.7	30	39.5	26	32.9	10	21.3
*51 to 65*	76	37.6	23	30.3	27	34.2	26	55.3
*65 plus*	40	19.8	16	21.1	13	16.5	1	23.4
**State**								
*New South Wales*	65	32.3	26	34.2	30	36.1	22	42.3
*Victoria*	31	15.4	9	11.8	14	17.9	8	17.0
*Queensland*	64	31.8	28	36.3	29	37.2	3	10.9
*Other states*	41	20.4	13	17.1	17	21.8	11	23.4
**Practice Setting**								
*Solo private practice*	137	67.8	51	67.1	51	64.6	35	74.5
*Group practice*	65	32.2	25	32.9	28	35.4	12	25.5
**Years of practice**								
*Less than 10 years*	51	25.2	19	25.0	23	29.1	9	19.1
*11 to 20*	72	35.6	27	35.5	31	39.2	14	29.8
*21 to 30*	48	23.8	19	27.7	16	20.3	13	27.7
*31 plus*	31	15.3	11	14.5	9	11.4	11	23.4
**Additional qualification**								
*None*	94	46.5	30	39.5	48	60.8	16	34.0
*Education*	39	19.3	16	21.1	12	15.2	11	23.4
*Complementary Medicine*	35	17.3	15	19.7	9	11.4	11	23.4
*Non-health*	30	14.9	12	15.8	8	10.1	10	21.3
*Health*	18	8.9	6	7.9	8	10.1	4	8.5

More than half of the participants (55.5%) had attained additional qualifications beyond their psychology qualification(s) in one or more of the following fields: education (n = 39), complementary medicine (n = 35), non-health related qualifications (n = 30), and health related qualifications (n = 18).

Psychologists estimates of what percent of their clients use CM ranged from 0% to 100%, with an average of 53% (*M* = 53.52, *SD* = 19.42). Psychologists were also asked how frequently they question their clients about possible CM use, with 82.6% (n = 166) reporting ‘most of the time’, 12.9% (n = 26) ‘sometimes’, and 4.5% (n = 9) ‘rarely or never’.

### Psychologists recommending CM products and/or practices

Mind/body approaches were the most recommended type of CM products and/or practices (90.5%) and cultural/spiritual approaches the least recommended (7.5%). Table 2 reports the results of the analysis conducted between demographic and practice characteristics and recommending types of CM. Psychologists holding additional qualifications in education (OR = 0.28 [0.11; 0.75]) or complementary medicine (OR = 9.33 [1.22; 1196.31]) were more likely to recommend mind/body approaches to their clients.

**Table 2:** Characteristics and recommending CM. Demographic and practice characteristics of psychologists and recommending CM products and/or practices, with odds ratios (OR) for recommending a specific product/practice, and a test (p, likelihood ratio test) of heterogeneity in probability of recommending a specific product/practice.

	Mind/Body (n = 202)		*p*	Movement (n = 202)		*p*	Ingestibles (n = 202)		*p*	Dietary changes (n = 201)		*P*	Manual (n = 202)		*p*	Cultural/ Spiritual (n = 201)		*p*
	OR	95% CI	LR	OR	95% CI	LR	OR	95% CI	LR	OR	95% CI	LR	OR	95% CI	LR	OR	95% CI	LR
**Gender**			0.30			0.99			0.24			0.97			0.48			0.45
*Female*	0.34	0.03; 1.4		0.97	0.37; 2.25		0.56	0.26; 1.67		0.90	0.38; 1.98		1.55	0.74; 3.22		2.26	0.52; 21.14	
*Male*	ref																	
*Other*	0.13	0.003; 21.6		0.76	0.03; 115.93		1.72	1.08; 258.78		1.03	1.05; 156.49		2.16	0.11; 324.41		7.88	0.04; 256.46	
**Age**			0.84			0.63			0.69			0.20			0.17			0.79
*18 to 35*	ref																	
*36 to 50*	1.51	0.25; 6.89		1.17	0.35; 3.51		1.65	0.61; 4.61		0.42	0.10; 1.35		0.61	0.20; 1.67		0.53	0.10; 3.25	
*51 to 65*	1.25	0.21; 5.19		1.83	0.54; 5.60		1.81	0.68; 4.97		0.72	0.17; 2.6		1.32	0.43; 3.71		0.79	0.19; 4.55	
*65 plus*	0.87	0.14; 4.04		1.17	0.33; 3.90		1.62	0.56; 4.83		0.36	0.08; 1.25		0.81	0.25; 2.47		0.48	0.6; 3.34	
**State**			0.67			0.64			0.80			0.68			0.78			0.18
*NSW*	ref																	
*VIC*	0.44	0.11; 1.85		0.55	0.21; 1.49		.97	0.41; 2.27		0.94	0.37; 2.45		1.13	0.46; 2.83		1.17	.003; 1.62	
*QLD*	0.56	0.15; 1.86		0.98	0.40; 2.36		1.16	0.58; 2.33		0.92	0.43; 1.98		0.97	0.47; 1.99		1.65	0.54; 5.45	
*Other states*	0.61	0.14; 2.49		0.92	0.35; 2.51		0.79	0.36; 1.71		1.56	0.63; 4.11		1.46	0.64; 3.49		0.69	0.12; 3.05	
**Practice setting**			0.62			0.48			0.38			0.38			0.87			0.11
*Solo practice*	ref																	
*Group practice*	1.29	0.48; 3.93		0.77	0.38; 1.59		1.29	0.72; 2.53		0.74	0.39; 1.44		0.95	0.51; 1.78		0.36	0.7; 1.24	
**Years of practice**			0.74			0.23			0.07			0.87			0.54			0.06
*Less than 10*	ref																	
*11 to 20*	0.63	0.14; 2.24		2.3	0.95; 5.74		2.60	1.26; 5.84		1.19	0.53; 2.64		1.68	0.80; 3.58		13.57	0.80; 3.58	
*21 to 30*	0.47	0.10; 1.77		1.12	0.46; 2.76		1.96	0.89; 4.39		1.42	0.58; 3.52		1.38	0.62; 3.51		13.02	1.41; 1728.48	
*31 plus*	0.59	0.11; 2.94		1.86	0.64; 6.03		2.01	0.82; 4.98		1.03	0.39; 2.78		1.67	0.66; 4.39		8.73	0.68; 1220.48	
**AoPE** ^1^			0.22			0.97			0.11			0.30			0.24			0.08
*General*	ref																	
*Clinical*	2.3	0.77; 8.39		0.97	0.44; 2.12		0.52	0.27; 0.98		0.65	0.32; 1.30		1.18	0.62; 2.81		0.29	0.07; 0.93	
*Other*	0.89	0.31; 2.74		0.90	0.37; 2.22		0.59	0.28; 1.23		1.15	0.49; 2.79		1.97	0.89; 4.54		0.34	0.06; 1.27	
**Additional qualifications**																		
*None*	1.51	0.59; 4.09	0.38	1.00	0.51; 2.00	0.98	0.65	0.37; 1.13	0.13	0.92	0.50; 1.73	0.81	1.01	057; 1.82	0.96	0.57	0.18; 1.63	0.30
*Education*	0.28	0.11; 0.75	0.01	0.79	0.36; 1.88	0.58	0.73	0.36; 1.47	0.38	0.51	.24; 1.06	0.07	0.65	0.32; 1.34	0.24	2.33	0.72; 6.77	0.14
*Complementary Medicine*	9.33	1.22; 1196.31	0.02	1.22	0.51; 3.32	0.17	2.58	1	1.21; 5.84	1.51	0.66; 3.85	0.34	1.77	0.80; 4.30	0.16	1.94	0.55; 5.86	0.28
*Non-health*	0.83	0.26; 3.32	0.76	0.75	0.32; 2.04	0.59	1.03	0.48; 2.25	0.92	0.77	0.33; 1.85	0.54	1.16	0.51; 2.75	0.71	1.04	0.19; 3.67	0.96
*Health*	0.43	0.13; 1.79	0.22	1.16	0.38; 4.63	0.80	1.43	0.55; 3.93	0.46	0.83	0.30; 2.58	0.73	1.25	0.46; 3.84	0.66	1.92	.035; 7.13	0.40

^1^Area of Practice Endorsement

### Psychologists referring to CM practitioners

Psychologists also refer to CM practitioners, with referrals to practitioners who prescribe ingestible products (e.g., naturopaths) the most common (57.9%), and referrals to cultural/spiritual healers/practitioners (e.g., Aboriginal and Torres Strait Islander traditional healer) the least common (6.9%). The results of Firth-corrected logistic regression yielded some interesting results for psychologists referring to CM practitioner types (see Table 3). While psychologists with 31 plus years experience were more likely (OR = 3.14 [1.27, 8.16]) to refer to manual therapy practitioners (e.g., massage therapists), those who were 65 plus years old were less likely to refer to movement therapy practitioners (OR = 0.93 [0.31, 2.79]) (e.g., personal trainer) indicating rates of referral were similar, with no evidence for difference.

**Table 3:** Characteristics and referring to CM. Demographic and practice characteristics of psychologists and their referring to types of CM practitioners with odds ratios (OR) for referring to a CM practitioner for a specific product/practice, and a test (p, likelihood ratio test) of heterogeneity in probability of referring to CM practitioner according to each of the characteristics.

	Mind/Body (n = 202)		*p*	Movement (n = 202)		*p*	Prescribes ingestibles (n = 202)		*p*	Prescribes nutrition (n = 201)		*p*	Manual (n = 202)		*p*	Cultural/ Spiritual (n = 201)		*p*
	OR	95% CI	LR	OR	95% CI	LR	OR	95% CI	LR	OR	95% CI	LR	OR	95% CI	LR	OR	95% CI	LR
**Gender**			0.81			0.66			0.63			0.81			0.22			0.48
*Female*	0.76	0.35; 1.77		1.19	0.58; 2.46		1.01	0.48; 2.08		0.91	0.44; 1.88		0.55	0.24; 1.14		2.10	0.48; 19.72	
*Male*	ref																	
*Other*	0.84	0.005; 17.13		0.37	0.002; 7.44		0.24	0.001; 4.82		-	-		0.21	.001; 4.31		7.88	0.04; 256.46	
**Age**			021			0.04			0.60			0.88			0.02			0.86
*18 to 35*	ref																	
*36 to 50*	2.69	0.57; 25.97		1.30	0.48; 3.62		1.63	0.61; 4.47		1.17	0.43; 3.20		3.06	1.03; 10.79		0.53	0.10; 3.25	
*51 to 65*	5.7	1.32; 53.57		2.50	0.94; 6.92		1.94	0.73; 5.27		1.30	0.49; 3.52		5.02	1.72; 17.53		0.68	0.15; 3.95	
*65 plus*	6.61	1.41; 64.19		0.93	0.31; 2.79		1.72	0.59; 5.10		1.48	0.51; 4.39		3.85	1.21; 14.33		0.49	0.07; 3.42	
**State**			0.38			0.19			.89			.64			0.84			0.18
*NSW*	ref																	
*VIC*	1.92	0.75; 4.85		0.44	0.18; 1.06		1.37	0.57; 3.35		1.4	0.62; 3.51		0.85	0.36; 2.01		0.22	0.35; 2.00	
*QLD*	0.85	0.67; 1.95		0.67	0.33; 1.33		1.10	0.55; 2.20		1.41	.71; 2.83		0.80	0.40; 1.60		2.05	0.62; 7.47	
*Other states*	0.95	0.37; 2.36		1.06	0.48; 2.34		1.24	0.57; 2.77		0.98	0.44; 2.14		1.11	0.51; 2.44		0.86	0.14; 4.10	
**Practice setting**			0.87			0.37			0.09			0.16			0.42			0.82
*Solo practice*	ref																	
*Group practice*	0.94	0.46; 1.87		0.77	0.38; 1.59		0.76	0.42; 1.37		0.65	0.35; 1.18		0.78	0.43; 1.41		0.88	0.25; 2.63	
**Years of practice**			0.08			0.36			0.31			0.35			0.02			0.09
*Less than 10*	ref																	
*11 to 20*	2.01	0.81; 5.41		1.76	0.86; 3.66		1.87	0.90; 3.85		1.46	0.71; 3.01		1.81	0.89; 3.76		11.80	1.38; 1543.80	
*21 to 30*	1.87	0.68; 5.38		1.36	0.61; 3.02		1.64	0.74; 3.66		1.11	0.50; 2.47		0.96	0.43; 2.16		10.65	1.09; 1426.53	
*31 plus*	3.80	1.36; 11.33		1.94	0.80; 4.82		2.00	0.82; 5.05		2.15	0.87; 5.48		3.14	1.27; 8.16		12.65	1.16; 1723	
**AoPE** ^1^			0.04			0.001			0.24			0.11			0.04			0.08
*General*	ref																	
*Clinical*	0.64	0.29; 1.39		0.84	0.44; 1.58		0.74	0.39; 1.40		0.73	0.39; 1.38		1.04	0.55; 1.95		0.23	0.04; 0.84	
*Other*	1.87	0.85; 4.15		3.22	1.49; 7.28		1.40	0.66; 3.05		1.59	0.76; 3.40		2.40	1.14; 5.16		0.57	0.14; 1.92	
**Additional qualifications**																		
*None*	0.61	0.31; 1.18	0.14	0.69	0.39; 1.19	0.18	0.87	0.49; 1.52	0.62	0.82	0.47; 1.43	0.49	0.58	0.33; 1.01	0.05	0.32	0.08; 1.00	0.05
*Education*	1.15	0.50; 2.48	0.12	1.02	0.51; 2.06	0.005	0.71	0.35; 1.42	0.33	0.95	0.47; 1.92	0.89	0.99	0.49; 1.99	0.99	3.52	1.14; 10.45	0.03
*Complementary Medicine*	1.16	0.48; 2.57	0.12	1.55	0.75; 3.29	0.23	1.96	0.92; 4.43	0.08	1.67	0.80; 3.59	0.17	1.30	0.63; 2.70	0.47	1.45	0.35; 4.70	0.57
*Non-health*	2.24	0.96; 5.05	0.05	0.65	0.29; 1.41	0.28	1.18	0.54; 2.69	0.67	0.87	0.39; 1.96	0.75	0.98	0.44; 2.14	0.95	1.16	0.22; 4.19	0.83
*Health*	0.97	0.28; 2.76	0.96	1.55	0.59; 4.24	0.81	1.12	0.43; 3.05	0.82	1.61	0.59; 4.72	0.35	1.05	0.40	0.91	3.38	0.79; 11.63	0.09

^1^Area of Practice Endorsement

Psychologists in the group with an AoPE in clinical psychology were less likely than other participating psychologists to refer to mind/body (OR = 0.64 [0.85, 4.15]), movement (OR = 0.84 [1.49, 7.28]) and manual therapy (OR = 1.04 [1.14, 5.16]) practitioner types, indicating rates of referral were similar, with no evidence for difference. Further, dual qualified psychologists, in education, were more likely to refer to movement and cultural/ spiritual practitioners. While those psychologists with dual qualifications, in non-health (e.g., law) were more likely to refer to mind/body practitioners.

## Discussion

This is the first study to identify rates of recommending different categories of CM products and/or practices and referring to different types of CM practitioners amongst Australian psychologists in clinical practice. One important finding from this study is that many psychologists appear to be actively engaging with CM in their clinical practice with a vast majority of participants reporting that they question clients about their CM use (95.5%), while a very large majority recommend CM products and/or practices (90.5%), and more than half of the sample refer clients to CM practitioners (57.9%). Although it is important to stress that the sample in our study was self-selecting, it is reasonable to cautiously suggest these levels of CM engagement amongst psychologists may provide first empirical indication of widespread grass-roots CM engagement amongst psychologists in clinical practice in Australia.

This finding from our study is consistent with, and may well be influenced in part by, CM engagement identified amongst general practitioners (GPs) [63, 74–76]. Some GPs in Australia report a motivation to engage with CM as part of a patient-centred, whole person approach to health care, as well as to meet demand from client groups that are high CM users, such as those experiencing chronic illness and mental health problems [62, 77, 78]. Given CM use for mental health problems is high [14, 79, 80] and GPs are the main referral pathway for these clients to psychologists, it may be that GPs are partly facilitating the inclusion of CM in the treatment planning for those clients also consulting a psychologist. Apart from common legislation around health professionals referring to health professionals (e.g., maintaining client confidentiality) there is no legislation that explicitly discusses the types of referrals psychologists can provide. Perhaps in lieu of CM specific guidelines from psychology associations in Australia [51], psychologists extend upon GP inclusion of CM to subsequently engage with CM in their own clinical practice with clients. Further research is required to identify what motivates psychologist engagement with CM.

In addition, the present study identified close to 50% of psychologists refer clients to CM practitioners who prescribe nutrition and movement. This may also be influenced by the growth of lifestyle medicine for mental health care among GPs [81, 82] that include CM, such as evidence-based social, nutrition and movement approaches [15, 83–87]. Wider acceptance of these approaches among GPs may promote discussion with clients, and subsequently influence discussions between these patients and the psychologist (to whom the GP refers them), regarding the role of nutrition and movement as part of mental health care. However, the specific channels by which such lifestyle medicine options may be introduced into the clinical practice of psychologists—introduced by clients, by the psychologist or by other health professionals whom the client may be consulting—remains unclear and requires further investigation.

The results of our study suggest some psychologists are seeking additional training and formal qualifications in non-psychology disciplines, including CM. More than half (n = 53.5%) of the participants had additional qualifications, including education (n = 19.3%) and CM (n = 17.3%). This is an interesting finding given that the Psychology Board of Australia does not support practitioners holding dual qualifications or applying more than one qualification to care for the same client. Interestingly, in the current study, having an additional qualification in education was associated with recommending mind/body approaches and dietary changes as well as referring to cultural/spiritual practitioners. This finding may be partly explained by psychologists with additional qualifications in education being exposed to CM approaches relevant to student and teacher wellbeing, such as mindfulness, yoga, healthy eating, and positive psychology [88–90], indicating they have greater knowledge of these approaches including potential benefits to clients. Further, as part of their training and work experience in educational settings, psychologists with additional tertiary training in education may also be exposed to the importance of cultural practices to mental health and wellbeing [91, 92]. Research on why psychologists seek additional skills from other non-psychology disciplines may assist our understanding of psychologist engagement with CM as an adjunct to conventional psychology approaches and is a topic requiring further empirical examination.

The current study also found psychologists do not engage with all types of CM equally. Only a small minority of psychologists in our study indicated they engage with cultural/spiritual approaches and clinical psychologists specifically were the lowest proportion of the participating psychologists to engage with recommending or referring to cultural/spiritual approaches. Perhaps our finding, that there is a low rate of engagement with cultural/spiritual approaches, partly reflects the critique of psychology as presented by some as a hegemonic Westernised approach to mental health [28, 93–95], which potentially minimises the relevance of including cultural/spiritual approaches in client’s care. Furthermore, psychologists in Australia may be reluctant to engage with culturally relevant approaches following the introduction of Medicare items for psychologists in 2006. These items include limited approved psychological therapies that are deemed appropriate to apply to all clients *regardless of their ethnocultural background* [96]. Any deviation from the approved listed therapies may result in an adverse outcome for a psychologist if they are audited by Medicare [97, 98] and it may be that these circumstances, partly at least, explain the low rates of engagement with culturally relevant or spiritual mental health care approaches amongst our study sample. Ultimately, further investigation is needed to help understand and explain this interesting finding.

The current study also identified psychologists with an AoPE in clinical psychology as less likely to refer to mind/body, movement, and manual therapies. Perhaps there is something unique to clinical psychology training and supervisory programs that influences clinical practice orientations and subsequently reduces engagement with CM. For example, it has been suggested there is risk of a narrowing of the field of psychology, where clinical psychology tertiary programs focus too much on cognitive behavioural therapy (CBT) [99], and not enough on broader social and cultural influences on mental health, as well as the role of client preference and values that might include CM approaches [100–102]. The literature also reinforces CBT as the gold standard therapeutic approach for psychologists, and that any drift away from CBT, such as engaging with CM approaches, would be ineffective and potentially harmful to clients [103, 104]. It may be that clinical psychologists are influenced by their clinical psychologist peers. We know for comparison that GPs report their professional networks as influencing their clinical practice through staying abreast of treatment advances as well encouraging them to stay in their professional niche to feel more secure [105]. Similarly, an Australian study of early career psychologists reported factors including postgraduate coursework and peer supervision as highly impactful on their theoretical and clinical practice orientations [106]. Further research is needed to understand what influences clinical psychologist’s reduced engagement with CM in clinical practice. For now, we can only suggest possible explanations for why clinical psychologists are less likely to engage with CM in their clinical practice than other psychologists in the study, and further research should examine this particular topic in more detail.

### Strengths and limitations

This study is the first to focus on the rates of recommending and referring to CM amongst a diverse range of psychologists in clinical practice (e.g., in terms of years of experience, AoPE). Although the number of participants in the study was small, it is representative of the Australian psychology workforce according to current workforce demographics provided by the Psychology Board of Australia [68]. There is potential for bias in our research due to participants being self-selecting, which may have resulted in larger numbers of psychologists who have an interest in CM being drawn to participating in the study.

### Future research

Future research is required to identify and investigate what motivates CM engagement amongst psychologists in clinical practice. Questions internal to the ranks of psychology that require further attention include: how do psychologists justify their engagement with CM to their clients as well as to others in their profession; and what do psychologists perceive and experience as the core challenges and benefits of CM integration within their practice and the broader field of psychology? Similarly, with a focus beyond the psychology field, we also need to know more details regarding how different CM providers perceive and understand their role in the care of those with mental health issues who also consult a psychologist. There is also a need to examine the perceptions and experiences of clients receiving concurrent and/or co-ordinated mental health care from both CM practitioners and psychologists, such as any detrimental effects that may have occurred as a result of referrals to CM practitioners from psychologists.

## Conclusion

A large proportion of Australian psychologists report some form of engagement with CM in their clinical practice. Policy and education development focusing upon this area of grass-roots psychology practice may help ensure the care provided by all psychologists remains evidence-based, safe and optimal for their clients.

## Supporting information

S1 Table Rates of recommending. Percentage of psychologists who recommend CM products and practices according to demographic and practice characteristics of psychologists and their recommending CM products and/or practices.
(DOCX)

S2 Table Rates of referring. Percentage of psychologists who refer to CM practitioners according to demographic and practice characteristics of psychologists and their recommending CM products and/or practices.
(DOCX)
