# Supplementary material for: The relationship between psychology practice and complementary medicine in Australia: Psychologists’ demographics and practice characteristics regarding type of engagement across a range of complementary medicine modalities
Source: PLoS One. 2023 May 4;18(5):e0285050. doi: 10.1371/journal.pone.0285050 (PMC10159172; doi:10.1371/journal.pone.0285050)
Supplement: S1 Table — Percentage of psychologists who recommend CM products and practices according to demographic and practice characteristics of psychologists and their recommending CM products and/or practices. (DOCX) [file pone.0285050.s001.docx]

Supplementary Table 1. Rates of recommending. Percentage of psychologists who recommend CM products and practices according to demographic and practice characteristics of psychologists and their recommending CM products and/or practices

|  | Mind/body  (n = 202) | Movement  (n = 202) | Ingestibles  (n = 202) | Dietary change  (n = 201) | Manual  (n = 202) | Cultural spiritual  (n = 201) |
| --- | --- | --- | --- | --- | --- | --- |
|  | n  (%) | n  (%) | n  (%) | n  (%) | n  (%) | n  (%) |
| **Total recommending** | 183  (90.5) | 161  (79.7) | 106  (52.4) | 147  (73.1) | 135  (66.8) | 15  (7.4) |
|  |  |  |  |  |  |  |
| **Gender** |  |  |  |  |  |  |
| *Female* | 147  (88.0) | 131  (78.4) | 82  (49.1) | 119  (71.2) | 113  (67.6) | 14  (8.3) |
| *Male* | 35  (97.2) | 29  (80.5) | 23  (63.8) | 27  (75.0) | 21  (58.3) | 1  (16.6) |
| *Other* | 1  (100.0) | 1  (100.0) | 1  (100.0) | 1  (100.0) | 1  (100.0) | 0  (0.0) |
| **Age** |  |  |  |  |  |  |
| *18 to 35* | 18  (90.0) | 15  (75.0) | 8  (40) | 17  (85.0) | 14  (70.0) | 2  (10.0) |
| *36 to 50* | 61  (92.4) | 51  (77.2) | 35  (53.0) | 45  (68.1) | 38  (57.5) | 4  (6.0) |
| *51 to 65* | 69  (90.7) | 64  (84.2) | 42  (55.2) | 59  (77.6) | 57  (75.0) | 7  (13.7) |
| *65 plus* | 35  (87.5) | 31  (77.5) | 21  (52.5) | 26  (65.0) | 26  (65.0) | 2  (10.0) |
| **State** |  |  |  |  |  |  |
| *NSW* | 61  (93.8) | 53  (81.5) | 34  (52.3) | 46  (70.7) | 42  (64.6) | 5  (7.6) |
| *VIC* | 31  (87.0) | 22  (70.9) | 16  (51.6) | 22  (70.9) | 21  (67.7) | 0  (0.0) |
| *QLD* | 57  (89.0) | 52  (81.25) | 36  (56.2) | 45  (70.3) | 41  (64.0) | 8  (12.5) |
| *Other states* | 37  (90.2) | 33  (80.4) | 19  (46.3) | 33  (80.4) | 30  (73.1) | 2  (4.8) |
| **Practice setting** |  |  |  |  |  |  |
| *Solo* | 123  (89.7) | 111  (81.0) | 69  (50.3) | 102  (74.4) | 92  (67.1) | 13  (9.4) |
| *Group* | 60  (92.3) | 50  (76.9) | 37  (56.9) | 45  (69.2) | 43  (66.1) | 2  (3.0) |
| **Years of practice** |  |  |  |  |  |  |
| *Less than 10* | 48  (94.1) | 37  (72.5) | 19  (37.2) | 35  (68.6) | 30  (58.8) | 0  (0.0) |
| *11 to 20* | 65  (90.2) | 62  (86.1) | 44  (61.1) | 53  (86.8) | 51  (70.8) | 8  (11.1) |
| *21 to 30* | 42  (87.5) | 36  (75.0) | 26  (54.1) | 37  (77.0) | 32  (66.6) | 5  (10.4) |
| *31 plus* | 28  (90.3) | 26  (83.8) | 17  (54.8) | 22  (70.9) | 22  (70.9) | 2  (6.4) |
| **AoPE** |  |  |  |  |  |  |
| *General* | 67  (88.1) | 61  (80.2) | 47  (61.8) | 57  (75.0) | 47  (61.8) | 10  (13.1) |
| *Clinical* | 75  (94.9) | 63  (79.7) | 36  (45.5) | 53  (67.0) | 52  (65.8) | 3  (3.7) |
| *Other* | 41  (87.2) | 37  (78.7) | 23  (48.9) | 37  (78.7) | 36  (76.5) | 2  (4.2) |
| **Additional qualifications** |  |  |  |  |  |  |
| *None* | 87  (92.5) | 75  (79.7) | 44  (46.8) | 68  (72.3) | 63  (67.0) | 5  (5.3) |
| *Education* | 31  (79.4) | 30  (76.9) | 18  (46.1) | 24  (61.5) | 23  (58.9) | 5  (12.8) |
| *Complementary medicine* | 35  (100.0) | 29  (82.8) | 25  (71.4) | 28  (80.0) | 27  (77.1) | 4  (11.4) |
| *Non-health* | 27  (90.0) | 23  (76.7) | 16  (53.3) | 20  (69.0) | 21  (70.0) | 2  (6.7) |
