# Supplementary material for: The relationship between psychology practice and complementary medicine in Australia: Psychologists’ demographics and practice characteristics regarding type of engagement across a range of complementary medicine modalities
Source: PLoS One. 2023 May 4;18(5):e0285050. doi: 10.1371/journal.pone.0285050 (PMC10159172; doi:10.1371/journal.pone.0285050)
Supplement: S2 Table — Percentage of psychologists who refer to CM practitioners according to demographic and practice characteristics of psychologists and their recommending CM products and/or practices. (DOCX) [file pone.0285050.s002.docx]

Supplementary Table 2. Rates of referring. Percentage of psychologists who refer to CM practitioners according to demographic and practice characteristics of psychologists and their recommending CM products and/or practices

|  | Mind/body  (n = 202) | Movement  (n = 202) | Prescribes ingestibles (n = 202) | Prescribes nutrition  (n = 201) | Manual  (n = 202) | Cultural spiritual  (n = 201) |
| --- | --- | --- | --- | --- | --- | --- |
|  | n  (%) | n  (%) | n  (%) | n  (%) | n  (%) | n  (%) |
| **Total referring** | 48  (23.7) | 102  (50.4) | 117  (57.9) | 99  (49.2) | 98  (48.5) | 14  (6.9) |
|  |  |  |  |  |  |  |
| **Gender** |  |  |  |  |  |  |
| *Female* | 38  (23.1) | 85  (51.8) | 96  (58.5) | 82  (50.0) | 76  (46.3) | 13  (7.9) |
| *Male* | 10  (27.7) | 17  (47.2) | 21  (58.3) | 17  (47.2) | 22  (61.1) | 1  (2.7) |
| *Other* | 0  (0.0) | 0  (0.0) | SM | 0  (0.0) | 0  (0.0) | 0  (0.0) |
| **Age** |  |  |  |  |  |  |
| *18 to 35* | 1  (5.0) | 8  (4.0) | 9  (45.0) | 9  (45.0) | 4  (20.0) | 2  (1.0) |
| *36 to 50* | 11  (16.6) | 31  (46.9) | 38  (57.5) | 32  (48.4) | 30  (45.4) | 4  (6.0) |
| *51 to 65* | 23  (30.2) | 48  (63.1) | 47  (61.8) | 39  (51.3) | 44  (57.8) | 6  (7.89) |
| *65 plus* | 13  (32.5) | 15  (37.5) | 23  (57.5) | 21  (52.5) | 20  (50.0) | 2  (5.0) |
| **State** |  |  |  |  |  |  |
| *NSW* | 15  (23.0) | 37  (56.9) | 36  (55.38) | 30  (46.1) | 33  (50.7) | 4  (6.1) |
| *VIC* | 11  (35.4) | 11  (35.4) | 19  (61.2) | 17  (54.8) | 14  (45.1) | 0  (0.0) |
| *QLD* | 13  (20.3) | 30  (46.8) | 37  (57.8) | 35  (54.6) | 29  (45.3) | 8  (12.5) |
| *Other states* | 9  (21.9) | 24  (58.5) | 25  (60.9) | 19  (46.3) | 22  (53.6) | 2  (4.8) |
| **Practice setting** |  |  |  |  |  |  |
| *Solo* | 33  (24.2) | 72  (52.9) | 82  (60.2) | 73  (53.6) | 69  (50.7) | 10  (7.3) |
| *Group* | 15  (23.0) | 30  (46.1) | 35  (53.8) | 28  (43.0) | 29  (44.6) | 4  (6.1) |
| **Years of practice** |  |  |  |  |  |  |
| *Less than 10* | 7  (13.7) | 21  (41.1) | 24  (47.05) | 22  (43.1) | 20  (39.2) | 0  (0.0) |
| *11 to 20* | 18  (25.0) | 40  (55.5) | 45  (62.5) | 38  (52.7) | 39  (54.1) | 7  (9.7) |
| *21 to 30* | 11  (23.4) | 23  (48.9) | 28  (59.5) | 22  (46.8) | 18  (38.2) | 4  (8.5) |
| *31 plus* | 12  (38.7) | 18  (58.0) | 20  (64.5) | 19  (61.2) | 21  (67.7) | 3  (9.6) |
| **AoPE** |  |  |  |  |  |  |
| *General* | 18  (23.6) | 35  (46.0) | 45  (59.2) | 38  (50.0) | 33  (43.4) | 9  (11.8) |
| *Clinical* | 13  (16.4) | 33  (41.7) | 41  (51.8) | 34  (43.0) | 35  (44.3) | 2  (2.5) |
| *Other* | 17  (36.9) | 34  (73.9) | 31  (67.3) | 29  (63.0) | 30  (65.2) | 3  (6.5) |
| **Additional qualifications** |  |  |  |  |  |  |
| *None* | 18  (19.1) | 43  (45.7) | 53  (56.4) | 45  (8.4) | 39  (41.5) | 3  (3.2) |
| *Education* | 10  (25.6) | 20  (51.3) | 20  (51.3) | 19  (50.0) | 19  (48.7) | 6  (51.4) |
| *Complementary medicine* | 9  (25.7) | 21  (60.0) | 25  (71.4) | 21  (61.8) | 19  (54.3) | 3  (8.6) |
| *Non-health* | 11  (37.9) | 12  (41.4) | 18  (62.1) | 13  (48.1) | 14  (48.3) | 2  (6.9) |
| *Health* | 4  (22.2) | 11  (31.1) | 11  (61.1) | 10  (62.5) | 9  (50.0) | 3  (16.7) |
